# Intestinal cytochromes P450 regulating the intestinal microbiota and its probiotic profile

**DOI:** 10.3402/mehd.v23i0.18370

**Published:** 2012-09-07

**Authors:** Eugenia Elefterios Venizelos Bezirtzoglou

**Affiliations:** Laboratory of Microbiology, Biotechnology and Hygiene, Department of Food Science, Faculty of Agricultural Development, Democritus University of Thrace, Orestiada, Greece

**Keywords:** intestinal cytochromes, P450, intestine, microflora, probiotic

## Abstract

Cytochromes P450 (CYPs) enzymes metabolize a large variety of xenobiotic substances. In this vein, a plethora of studies were conducted to investigate their role, as cytochromes are located in both liver and intestinal tissues. The P450 profile of the human intestine has not been fully characterized. Human intestine serves primarily as an absorptive organ for nutrients, although it has also the ability to metabolize drugs. CYPs are responsible for the majority of phase I drug metabolism reactions. CYP3A represents the major intestinal CYP (80%) followed by CYP2C9. CYP1A is expressed at high level in the duodenum, together with less abundant levels of CYP2C8-10 and CYP2D6. Cytochromes present a genetic polymorphism intra- or interindividual and intra- or interethnic. Changes in the pharmacokinetic profile of the drug are associated with increased toxicity due to reduced metabolism, altered efficacy of the drug, increased production of toxic metabolites, and adverse drug interaction. The high metabolic capacity of the intestinal flora is due to its enormous pool of enzymes, which catalyzes reactions in phase I and phase II drug metabolism. Compromised intestinal barrier conditions, when rupture of the intestinal integrity occurs, could increase passive paracellular absorption. It is clear that high microbial intestinal charge following intestinal disturbances, ageing, environment, or food-associated ailments leads to the microbial metabolism of a drug before absorption. The effect of certain bacteria having a benefic action on the intestinal ecosystem has been largely discussed during the past few years by many authors. The aim of the probiotic approach is to repair the deficiencies in the gut flora and establish a protective effect. There is a tentative multifactorial association of the CYP (P450) cytochrome role in the different diseases states, environmental toxic effects or chemical exposures and nutritional status.

In the recent past, the study and understanding of the intestinal microecology, its metabolic capacity, and its regulation has expanded enormously. A part of its metabolic capacity seems to be in close relation with the presence of the cytochromes on the small intestine. The cytochrome P450 (CYP) constitutes a superfamily of hemoproteins that catalyze both endobiotic and xenobiotic substances. Xenobiotics include commonly used medicines, which will be appropriately metabolized to varying degrees by the aid of cytochromes. Their catalyzing abilities are known for many chemical reactions such as hydroxylation, epoxidation, or heteroatom oxidation. Their endogenous function includes biosynthesis of steroids, bile acids, and vitamin D3 and metabolism of fatty acids, prostaglandins, biogenic amines, and retinoids. Their exogenic function concerns drug metabolism and disposition, pharmaceutical substances, antioxidants, environmental and chemical residues, and finally probiotics disposition. However, as the role of the intestinal CYP was not extensively studied, we focused on specifically reviewing the expression of CYP present in the small intestine as considerable amounts of the different cytochromes are found on it. In particular, CYP3A represents an average content of approximately 80% of spectrally determined P450 followed by CYP2C9 (15%). The bacterial species needed for inducing a proper barrier effect is not known, but it is generally accepted that this barrier function can be strongly supported by providing benefic alimentary supplements called functional foods. Herein, the fact that early intestinal colonization with organisms, such as lactobacilli and bifidobacteria, would possibly protect from many different types of diseases is emphasized. Moreover, this beneficial microflora dominated by bifidobacteria and lactobacilli lends credence to their ability to modify the gut microbiota as their ability to decrease β-glucoronidase and carcinogen levels reduces the risk of cancer.

## Biochemical status and role

CYP isoenzymes belong to the large family of hemoprotein enzymes, which are the terminal oxidases of the mixed function oxidase system found on the membrane of the endoplasmic reticulum (ER). They are also found in the inner mitochondrial membrane.

All biological organisms such as plants, animals, bacteria, fungi, and humans possess P450 isoenzymes. A common ancestral gene seems to be the origin of the superfamily as the extensive similarity between CYP identified in man and bacteria date back to more than 3.5 billion years. There is a particularly interesting theory that the P450 xenobiotic-metabolizing enzymes were formed following a continuous survival fight between plants and animals (1, 2). The evolutionary step seems to link plant to animal from around 2.3 or 2.4 billion years ago. Plants have to protect themselves from animal predators by synthesizing toxic metabolites. As result, animals have developed specific enzymes to compete against plant toxins (1).

However, research on cytochromes originated from different species has always been done with the view of application to humans, including the research performed on the cytochromes of other living organisms.

The CYP proteins with more than 55% identity are grouped under the subfamily, indicated by a capital alphabetic letter, the Arabic number following this letter expresses the individual enzyme, and its associated gene is denoted in italics (e.g., *CYP27A2* or *CYP27A2*).

To date, a total of 270 CYP gene families have been found. Plants carry many cytochromes P450 CYP enzymes and then produce a huge array of chemicals, hence, metabolomics are of great importance in plant ecology. In particular a metabolome which is a complete set of small molecules in an organism has traits to reveal the many loci responsible for the regulation enzyme activity.

In humans, more than 74 CYP genes and 33 pseudogenes arranged into 18 families and 42 subfamilies have been found (3, 4). CYP develops spectral peak at near 450 nm because of the presence of a thiol group, which serves as a ligand for the heme–iron.

Researchers based on these spectral properties nominated so far these isoenzymes P450. CYP proteins in all species are classified on the basis of their amino acid sequence. Proteins with more than 40% identity are grouped under the same family, indicated by an Arabic number. The prefix CYP designates cytochrome P450 isoenzymes in all species (5, 6).

## Role

P450 isoenzymes play a vital role in the metabolism of both endogenous and exogenous substances. Many substances are metabolized to varying degrees by different P450s as a result of their large broad substrate specificity.

Endogenous function includes biosynthesis of steroids, bile acids, vitamin D3 and metabolism of fatty acids, prostaglandins, biogenic amines and retinoids. Exogenic function concerns itself with drug metabolism and disposition, pharmaceutical substances, antioxidants, environmental and chemical residues, and finally probiotics disposition by producing metabolic end-products, which could be toxic or carcinogenic in several cases.

Moreover, CYP is believed to have a role in the hepatic drug detoxication system. During the past decades, with the development of recombinant DNA technology and advances in mRNA purification, cDNAs encoding the complete human CYP protein have been isolated, and thereafter, results of many cloning studies have revealed many different enzymes. However, most research was focused on their role in different disease states.

## Classification

CYPs are divided into four classes on the basis of how electrons from NAD(P)H are delivered to the catalytic site (5, 6):

1. Class I proteins require both an FAD-containing reductase and an iron sulfur ferredoxin for transfer of electron.
2. Class II proteins require only an FAD/FMN-containing P450 reductase for transfer of electron.
3. Class III proteins are self-sufficient and do not require electron donor or molecular oxygen for catalyzing hydroperoxides.
4. Class IV proteins receive electrons directly from NAD(P)H.

## Structural features

P450 isoenzyme core structure comprises a single protein built around a central heme group. Sequence identity among P450 enzymes is low (<20%), as only three amino acids are well conserved.

The core structure is constant in the proteins that are responsible for the mechanism of electron/proton exchange leading to oxygen activation. This constant core consists of four-helix bundle (D, E, I, and L), two helices J and K, two sets of β sheets, and the ‘meandler’ (5, 6).

A heme-binding loop, a characteristic domain with a conserved cysteine amino acid, serves as a ligand to the heme iron. Another well-conserved domain is found in the helix K on the proximal side of the heme iron, which is necessary to stabilize the core structure (7).

Moreover, the central part of the helix I has a conserved amino acid sequence, which is responsible for the proton transfer on the distal end of the heme. Variable flexible regions involve in many functions, such as substrate binding, amino terminal anchoring, and targeting membrane-bound proteins (5, 6, 8).

## Biochemical function

Hydrophilic or polar metabolized products are formed at the end of the reaction, catalyzed by P450. The products can be excreted easily in this form. Microsomal CYP requires an additional enzyme called flavoprotein cytochrome P450 reductase to transfer electrons from NADPH to CYP. Bacterial and mitochondrial CYP requires two additional proteins (5, 6, 8):

1. Adrenodoxin reductase, a flavoprotein which involve in the electron transfer mechanism from NADPH to the iron–sulfur protein.
2. Adrenodoxin, which reduces CYP.

The overall reaction catalyzed by CYPs is as follows:

$$\text{NADPH +}{\text{H}}^{\text{+}}\text{+}{\text{O}}_{\text{2}}\text{+RH}\to \text{NAD}{\text{P}}^{\text{+}}\text{+}{\text{H}}_{\text{2}}\text{O+R-OH,}$$

where R represents a substrate that serves as a site of oxygenation. However, CYP drug metabolization develops distinct substrate specificity. It is also important to note that there is considerable interindividual variation in the expression and activity of CYP in the human liver.

Many studies report drug interaction in patients that involve induction or inhibition of CYP (9–11). These studies have focused on the importance of genetic polymorphism that involves P450 isoenzymes (9).

Lacking of functional P450 forms in CYP2C19 and CYP2D6 subfamilies is observed in many patients (12–15). These intra- and interindividual variations are extrapolated to ethnic ones, as several polymorphisms may be prevalent in specific ethnic groups or population. It would thus make sense to focus attention on adverse drug reactions among a certain ethnic or patient group (16).

Sexual variation seems to be effective as well (17). In the course of early embryogenesis, during sexual differentiation of the genital ridge, the transcriptional steroid factor 1 is submitted in regulation of P450 genes implicated in the synthesis of steroid hormones, such as CYP11, CYP17, CYP19, and CYP21 families. The CYP11 enzymes are mitochondrial enzymes. The CYP17 enzymes are located on the ER and are involved in the synthesis of adrenal cortical hormone, testosterone, and estrogen, whereas the CYP19 enzymes are designated to transform the androgenic precursors into estrogens.

CYP isoenzymes follow the principle of Michaelis–Menten kinetics and are dependent on different cofactors, such as glucocorticoids (CYP3A) and polycyclic hydrocarbons (CYP1A) for their induction or inhibition (1, 5, 6, 18). This activation of the P450 enzyme is effective under different mechanical pathways: heme direct binding, heme indirect binding *via* oxidative substances, direct irreversible inactivation, or competition for substrates.

Besides their important role in drug metabolism, P450 enzymes have a functional role in other tissues due to their ubiquity, where they develop regulated patterns of expression. A plethora of pharmaco-toxical studies of P450 enzymes and genes concern the field of drug metabolism and adverse drug reaction focusing on P450 expression in the hepatic tissue. In addition to the liver, the P450 cytochromes also play vital role in lung, kidney, pancreas, brain, adrenal gland, bone marrow, skin, mast cells, testis, ovaries, olfactory organs, and small intestinal mucosa. Despite the low overall level of CYP enzymes (∼1%) in the small intestine, they are an important extrahepatic site of drug metabolism. As the role of CYP in the intestine was less extensively studied, we focused to specifically determine the expression of CYP in the small intestine.

## Expression of CYP in the intestine

### Intestinal system distribution

Despite the fact that the intestinal system is part of the human body's first line of defense against orally ingested xenobiotica, little is known about the distribution and expression of CYP enzymes in human intestine.

Therefore, expression and protein levels of the different CYPs have to be determined by relevant methodologies, such as RT-PCR or Western blot methods, for determining protein concentration (%) of CYPs. The small intestine is an important site of first-pass metabolism of numerous drugs, food components, and toxic xenobiotics. However, there is not much information available about age-dependent changes of intestinal biotransformation pathways (19).

**Fig. 1:**
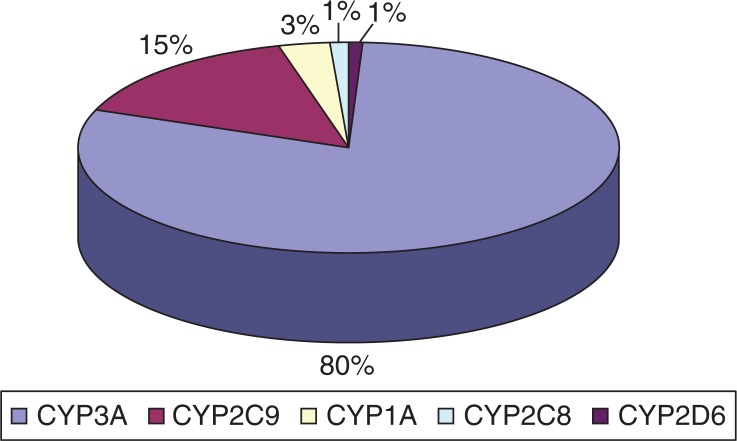
Distribution of P450 intestinal cytochromes (protein concentration of CYPs is determined in %). Sources: Collection of bibliographic information and data Ref. 3, 18, 21, 23.

The most common P450 cytochrome subfamily expressed in the mucosa of the small intestine is CYP3A, which represents an average content of approximately 80% of spectrally determined P450 content, followed by CYP2C9 (15%) (Fig. 1).

CYP1A cytochrome is expressed in the duodenum, together with less abundant levels of CYP2C8-10 and CYP2D6.

### Genetic polymorphism

As discussed, there is a genetic polymorphism intra- or interindividual and intra- or interethnic. The CYP2D6 enzyme is associated with genetic polymorphism as mutation of the gene CYP2D6 located on chromosome 22 can result in reduced affinity forms of the enzyme.

Caucasians (5–10% of the total population) and Asians (0.9%) show decreased metabolization rates of debrisoquine. However, it must be noted that there is a group of ultra-rapid metabolizers associated with CYP2D6, which results from gene amplification (9). Moreover, it seems that the activity of CYP complex in rat small intestine was not decreased by the aging process, so the high rate of oxidative metabolic reactions in intestinal mucosa can be maintained till the advanced life stage (19).

### Pharmacokinetic impact and functional role

Bioconversion within the intestinal system is important because it decreases drug bio-availability after oral administration (20–23).

Changes in the pharmacokinetic profile of the drug are associated with increased toxicity because of reduced metabolism, altered efficacy of the drug, increased production of toxic metabolites, and adverse drug interaction (24–26).

The functional role of the most common CYP families expressed in the intestine stimulated our interest.

## The CYP1A cytochrome family

This family concerns two subfamilies, which are CYP1A and CYP1B (2). The *CYP1A1* and *CYP1A2* genes are found on chromosome 15 and have been fully characterized and associated to the aryl hydrocarbon receptor (1, 18, 27), which could be activated by binding of polycyclic aromatic hydrocarbons found in cigarette smoke, charcoal-grilled food, and incineration procedures.

The CYP1A2 encounters additional roles of inactivating prostaglandin G_2_, hydroxylating estrogen, oxidizing uroporphyrinogen and melatonin (3, 9). This latter cytochrome CYP1A2 is reported to be involved in metabolic procedures of more than 20 different drugs (22), whereas CYP1A1 and CYP1B1 do not confer mainly in metabolizing drugs.

CYP1B1 is expressed in many tissues, including the intestines. This newly characterized cytochrome is implicated in metabolizing endogenous estrogens. However, it keeps an important role in the transformation of heterocyclic amines involved in charcoal-broiled meat (28).

## The CYP2 family

The CYP2 family is the largest P450 family in mammals (22). This family encompasses many subfamilies, such as CYP2A, CYP2B, CYP2C, CYP2D, and CYP2E (5).

Both CYP2A and CYP2B subfamilies consist of a plethora of different members identified in various species (29) and expressed at low levels in humans, specifically the human liver.

Our focus was on members of the CYP2C and CYP2D subfamilies, which are expressed in the human duodenum. Generally, low levels of CYP2C8, CYP2C 10, and CYP2D6 are found in the human duodenal tissues (4). However, CYP2C9 is expressed in significant levels (15% of the spectrally determined P450 content) in the mucosa of the human small intestine and also the liver (17–20%) (30). Enzymes of this CYP2A subfamily are responsible for the metabolism of a large range of usually prescribed pharmaceutical agents, such as *S*-mephenytoin, phenytoin, *S-*warfarin, tolbutamide, arachidonic acid, steroids, and non-steroidal anti-inflammatory substances (31, 32). Moreover, rifampin seems to induce effectively the CYP2C family members. Mephenytoin, tolbutamide, and phenytoin were proposed to measure *in vitro* the CYP2C activity.

As discussed, another subfamily expressed in the duodenum is the CYP2D, which is identified except for the human species in different other species. This subfamily includes five members, most of them expressed in the rat. In the human duodenum, CYP2D6 form is expressed in low levels (∼2%). Xenobiotics substrates with basic nitrogen atoms metabolized by this P450s include debrisoquine, clozapine, codeine, ethyl morphine, dextromethorplan, spartein, perhexiline, and timolol. The enzyme doesn't seem to be inducible. Through these substrates, dextromethorphan was proposed for measurement of CYP2D activity and phenotyping individual species (3).

Recent studies indicate NNK to be a xenobiotic substrate for CYP2D6 expressed in the human liver or duodenum (27, 33). Inhibitors of CYP2D6 include quinidine, quinine, paroxetine, norfluoxetine, and fluoxetine.

Finally, the CYP2E subfamily included in this CYP2 major family does not seem to be involved in any intestinal expression; however, it is associated with other extra hepatic places such as the human respiratory tract (13, 34).

## The CYP3 family

This family is responsible for the metabolism of a wide brood of therapeutic agents (30).

At present, 22 different CYP3A subfamily members were identified in various species, including humans. It is an important P450 subfamily in the human liver, as it is associated with 40% of the total P450 in human liver. However, its abundance in the mucosa of the small intestine (70%) includes its key role within this tissue (35). CYP3A4 and CYP3A5 forms are expressed in the liver and in the intestinal mucosa (36). This latter CYP3A5 form seems to be extremely high in the human intestine (70%). Induction of CYP3A enzymes is effective usually by rifampin or phenobarbital followed by dexamethasone, phenytoin, and carbamazepine (37, 38). Induction specifically by CYP3A5 subfamily enzymes seems to be performed by rifampin or dexamethasone substrate applied for measurement of CYP3A activity include nifedipine, midazolam, testosterone-6β-hydroxylation. TAO, erythromycin, and gestodene are the main inhibitors of the CYP3A5 enzymes.

Clarithromycin, midazolam, ketoconazole, clotrimazole, naringenin, 6, 7-diOH-bergamottin, and the aforementioned agents are reported as inhibitors of the CYP3A4 subfamily. However, it is important to state that duodenal CYP3A4 absent in the total duodenum was found present in neonates and children in low levels (15). Active celiac disease was associated to low levels of CYP3A4 (12). It appears that unknown factors controlling maturational changes are implicated for differences in drug metabolism and disposition in the pediatric population (15) by the CYP3A4 intestinal enzymes.

A plethora of xenobiotic substrates medically important are metabolized (Table 1) by the CYP3A family enzymes. Relevant substrates of toxicological interest include 1-nitropyrene, 6-aminochrysene, and aflatoxin B1 (30, 36).

**Table 1:** The gastrointestinal P450 sites

Enzyme P450	Tissue involved	Xenobiotic substances transformation	Metabolism of endogenous substrates	Metabolism of xenobiotic substances				Induction	Inhibition
CYP1B1	Intestine	Herocyclic amines (charcoal meat) Arylarenes Nitroarenes	Testosterone 6β, 16α-hydroxylation 17β-Estradiol-C_4_-hydroxylation	7-Ethoxy-4-trifluoro methylcoumarin Ethoxyresorufin					
CYP2C8-10	Duodenum			Phenytoin Tolbutamide Retinol	*S*-methynytoin-4-hydroxylation Retinoic acid			Rifampin	Sulfaphenazol Sulfinpyrazonee
CYP2C9	Small intestine (15%) mucosa			Diclofenac Flubiprofen Mefenamic acid Chloramphenicol	Diflunisal Hexobarbital	Tolbutamide *S*-warfarin-7-hydroxylation Ibuprofen Mefenamic acid		Rifampin	Sulfaphenazole Warfarin Tolbutamide
				*S*-methenytoin-4-hydroxylation		Losartan	Omeprazole		
				Phenytoin	Naproxen	Tolsemide	Retinoids		
CYP2D6	Duodenum	NNK		Codeine Bufarolol Amitriptyline Deorisoquine Imipramine Mexiletine	Clozapine Metoprolol Nortriptyline Desipramine Odansetron Encainide	Dextromethorphan Ethylmorphine Propafenone Perhexiline Thioridazine Timolol	Flecainide Sparteine Perphenazine Tramadol		Quinidine Quinine Fluoxetine Norfluoxetine Paroxetine
CYP3A4	Small intestine mucosa (70%) Neonates duodenum	Acetaminophen Aflatoxin B1 Cyclophosphamide Ifosfamide Benzo[a]pyrene-7,8-dihydrodil	testosterone 6β, 2β, 6α, 18, 16β, 15β-hydroxylation Cortisol 6β hydroxylation, Androstenedione 6β-progesterone hydroxylation 17β-estradiol-2-hydroxylation	Carbamazepine Chloropromazine Clarithromycin Alfentamil Aminodarone Cyclosporine A Dexamethazone Diltiazem Verapamil R-warfarin Temazepam	Clozapine Erythromycin Antipyrine Dapsone Diazepam Fluvastatin Ethinylestradiol Zidovudine Triazolam Trimethoprim	Trimethadione Lidocaine Indinavir Nelfinavir Nifedipine Tomoxifen Paclitaxel Saquinavir Retinoic acid Quinine Proguanil	Imiprimod Lovastatin Midazolam Omeprazole Tacrolimuns Terfenadine Orphenadrine Simvastatin Quinidine Prednisone		Erythromycin Clarithromycin Gestodene Ketoconazole Midozolam Clotrimazole Oleandomycin TAO 6,7-diOH-bergamottin Naringenin
CYP3A5	Small intestine mucosa (70%)		Testosterone 6β, 2β-hydroxylation	Nifedipine Midazolam				Dexamethasone Rifampin	Erythromycin TAO Gestodene

## The CYP4 family

This family which consists of 11 subfamilies (39) is newly characterized and insufficiently studied. CYP4 family enzymes have not been proven to be expressed in any part of the intestine. Constructively, CYP enzymes in intestinal tissues play a dominant role in the metabolic activation of xenobiotic substrates (13). Moreover, the large intestine epithelium is recognized to possess receptors which set the host innate response.

Quorum-sensing between microorganisms of the gut flora and epithelial cells is guided *via* some specific receptors of the human gut such as nuclear receptors (NRs) or Toll-like receptors (TLRs) (21). Maturation and integrity of the intestinal epithelium is important not only for the above communication between the intestinal epithelium and microbiota and setting of the host innate response but also influences drug disposition and metabolism. Effects of age and disease states seem to be crucial on the expression and activity of the intestinal CYP3A4 and other P450s enzymes (12, 40). Duodenal, intestinal and gastric pH, intestinal transit time, gastric emptying time, P-glycoprotein, and bacterial colonization may influence drug absorption (15).

Differences in drug distribution could be explained by ageing which induce modifications of the circulating endogenous substances in plasma, total body fluids, extracellular water, fat, and protein concentration in plasma leading to another pattern of membrane permeability (15). Leaky gut syndromes are health disorders associated with increased intestinal permeability (14). These syndromes account for a broad range of conditions, such as inflammatory or infectious bowel diseases, extended to cryptogenic skin conditions or other states (14).

When rupture of the intestinal integrity occurs, increased passive paracellular absorption is observed. Moreover, an increase of oxygen radicals and carcinogens production is effective by the CYP mixed-function oxidation system (14). The importance of interindividual and sex-based differences in pharmacokinetics and pharmacodynamics is stated (17). Bioavaibility, drug metabolism, and distribution are influenced by a plethora of factors as reported previously considering the CYP activity (9).

However, it must be stated that the main role of the P450 enzymes are to convert lipophilic chemical substances called xenobiotics, in which human body surfaces or organs are exposed to more water soluble substances. It is like a detoxifying mechanism as these lipophilic substances could be harmful and toxic after being concentrated in our tissues.

Enzymes involved in xenobiotic biotransformation belong to phase I or phase II reactions (41). Toxic or tumor-induced effects are provoked by conversion of some xenobiotics to reactive electrophilic metalites forming DNA and protein adducts (2) in phase I. Phase I states basically an oxidation, reduction or hydrolysis reaction, while phase II focuses on conjugation of the xenobiotic compound with a molecule. Localization of the P450s in the intestinal and duodenal tissue is of crucial importance, as such extrahepatic metabolism balance eventually the reduced hepatic function following liver disease states (42).

Important biochemical events take place in the human intestine. To explain these biochemical functions of the gut, two terms were brought to light; MAC (microflora-associated characteristics) and GAC (germ-free animal characteristics) (43). The concept of MAC/GAC is based on disturbances in functions, such as conversion of cholesterol to coprostanol, conversion of bilirubin to urobilinogen, presence/absence of β-aspartylglycine, inactivation of tryptic activity. Absence of the above functions is observed in germfree animals, healthy newborns or sometimes in individuals receiving antimicrobial agents (44). Characteristic shifts in intestinal microflora of conventional or germfree animals are associated to induction or repression of certain isoforms of P450s (45). The reported high metabolic capacity of the intestinal flora seems to be based on its enormous pool of enzymes (1) that catalyze reactions in phase I (41, 43) and phase II (9). This high enzymatic activity of the intestinal microflora seems to be of interest for new research, as much scientific work (1, 18) focused on the presence of P450s in the major bacterial strains coming from the human fecal flora (46). Intestinal *Eubacterium aerofaciens* was found to have a CYP -like gene (46). *Desulfomonas pigra* isolated from human feces was found to have a cytochrome c and a desulfoviridin-like pigment (47). The genome of *Streptomyces coelicolor* A3 (2) revealed 18 cytosolic CYPs with six ferredoxin proteins and two soluble ferredoxin reductases (48, 49).

The C-type cytochromes (50) in *Geobacter sulfurreducens* are involved in electron transfer to Fe (III) oxides. *Eubacterium lentum* was shown to contain cytochromes a, b, and c and a carbon monoxide-binding pigment (51).

## The intestinal microbiota profile

Although, many tissues within the body are known to posses some CYP activity, the prevailing dogma is that the intestine is associated to an important extend with CYP metabolism, as it is responsible for the extra hepatic metabolism (52). It is clear that high microbial intestinal charge following intestinal disturbances (53), ageing, environment or food associated, leads to the microbial metabolism of a drug before absorption (54). Moreover, it is obvious that knowledge on the CYP system is of tremendous interest because of its key role in steroid hormone formation, carcinogen activation, and drug metabolism. Knowledge about drug absorption, metabolism and excretion, interaction with food or other drugs, suppression, enhancement, and antagonism of endogenous agents open more effective channels for clinicians to complete their therapeutic purposes. Based on the fact that many intestinal bacterial strains possess P450 enzymes, the question is raised that if live probiotics express a P450 activity, which of them could eventually influence the drug metabolism and bioavailabity? The human body has developed a holistic defense system, which mission is either to recognize and destroy the aggressive invaders or to evolve mechanisms permitting to minimize or restore the consequences of harmful actions. The host immune system keeps the capital role to preserve the microbial intestinal balance *via* the barrier effect. Specifically, pathogenic invaders such as bacteria, parasites, and viruses and other xenobiotic invaders are rejected out of the body *via* barriers formed by the skin, mucosa, and intestinal flora. In case those physical barriers are breached, the immune system with its many components comes into action to prevent infection. The intestine itself is considered as an ‘active organ’ due to its abundant bacterial flora and to its large metabolic activity. The variation among different species or even among different strains within a species reflects the complexity of the genetic polymorphism which regulates the immune system functions. Additionally, factors such as gender, particular habits, smoking, alcohol consumption, diet, religion, age, gender, precedent infections, and vaccinations must be involved (55). Hormonal profile and stress seems to be associated to the integrity microbiota and inducing immune system alterations. It is not known which bacterial species are needed for inducing a proper barrier effect, but it is generally accepted that this barrier function can be strongly supported by providing benefic alimentary supplements called functional foods. In this vein, it is stressed the fact that early intestinal colonization with organisms such as lactobacilli (56) and bifidobacteria provide possibly subsequent protection from many different types of diseases. Moreover, this benefic microflora dominated by bifidobacteria and lactobacilli supports the concept of their ability to modify the gut microbiota by reducing the risk of cancer following their capacity to decrease β-glucoronidase and carcinogen levels. Within a few hours from birth the newborn develops its normal bacterial flora. Indeed human milk frequently contains low amounts of non-pathogenic bacteria like *Streptococcus, Micrococcus, Lactobacillus, Staphylococcus, Corynebacterium*, and *Bifidobacterium* (55). In general, bacteria appear in feces within a few hours after birth. Colonization by *Bifidobacterium* occurs generally within 4 days of life. The effect of certain bacteria having a benefic action on the intestinal ecosystem is largely discussed during the last few years by many authors. *Bifidobacterium* is reported to be a probiotic bacterium, exercising a beneficial effect on the intestinal flora. An antagonism has been reported between *B. bifidum* and *C. perfringens* in the intestine of newborns delivered by caesarean section (56).

## Toxicity of products of CYP reactions

It was suggested that CYP2E1 may play a role in alcohol-induced liver damage (57). Malignant tumors of the upper gastrointestinal tract and liver are revealed from epidemiological data correlated closely to the consumption of ethanol. It is known that oxidants play a key role in alcohol-induced liver injury. CYP2E1 is required for the induction of oxidative stress to DNA, and thus may play a key role in ethanol-associated hepatocarcinogenesis (58).

The degradation of ethanol by CYP2E1, as the result of uncoupling of oxygen consumption on NADPH oxidation, produces ${\text{O}}_{2}^{-}$ and H_2_O_2_ that can deplete glutathione and cause cellular damage. When ethanol is degraded by CYP2E1, reactive oxygen species (ROS), which are powerful oxidants such as the hydroxyl radical (59, 60), are formed as by-products of the reaction, while ethanol itself is oxidized in a 1-electron reaction to form 1-hydroxyethyl radical.

Similar 1-electron oxidations take place with several other substrates of CYP2E1, but perhaps not all of them. Such organic radicals can initiate free-radical chain reactions that can lead to production of mutagenic products of polyunsaturated fatty acid peroxidation or react directly upon DNA acting as mutagens by themselves.

In this context, correlative analysis revealed that CYP2E1 is mainly focused in the perivenular area, where liver damage has occurred in alcohol-induced cirrhosis patients (57). However, most research was focused in ethanol toxicity in adults and specifically the most affected organs seem to be liver and brain.

Excessive alcohol consumption during pregnancy can result in serious adverse effects to the fetus called FAS. The fetal alcohol syndrome (FAS) is associated with growth retardation, central nervous system damage, and facial dysmorphology; this ethanol teratogenicity can affect the fetus during pregnancy because of excessive alcohol consumption (61).

It should be stated, that the alcohol-induced liver damage model in humans may differ from that in mice because of intraspecies differences in hepatic structure and architecture, as well as in differences in stress responses.

The degradation of estrogens by CYP1A1 and CYP1B1 and their oxidation to catecholestrogens (CEs) remain of capital interest to their catalytic importance in cancer initiation. CEs are among the major metabolites of E_1_ and E_2._

CEs formed function as redox-cycling agents producing ROS *in vivo*. However, when these metabolites are oxidized to catechol estrogen quinones (CE-Q), they may react with DNA to form depurinating adducts (62) leading to mutations which may initiate human cancers (breast, prostate).

Another example is the case of benzene when hydroxylated in two consecutive CYP-catalyzed reactions to form hydroquinone, or when 1, 4-dichlorobenzene is hydroxylated in two consecutive CYP-catalyzed reactions to form 1, 4-dihydroxy-2, 5- 1, 4-dihydroxy-2,5-dichlorobenzene (62). The ability of hydroquinone and 1,4-dihydroxy-2,5-dichlorobenzene to function as ROS-generating redox cycling agents depends on the relatively high stability of the organic radical formed, which in both cases is a semiquinone radical.

The estrogens are biochemically convertible by the enzyme 17-β-estradiol dehydrogenase and metabolized *via* two main pathways: formation of CE and 16-α-hydroxylation. The estrogens formed are the 2-hydroxylated and 4-hydroxylated CEs (63, 64), which can be inactivated by *O*-methylation catalyzed by catechol-*O*-methyltransferases (COMT) (63). These phase II enzymes are important not only for transforming xenobiotics into excretable products, but mainly for contributing to the detoxification of highly toxic metabolites. However, it must be noted that deficiency in folate or vitamin B12 (65) leading to decreased concentration of *S*-adenosylmethionine, which is used as methyl-donating substrate by COMT. If glutathione-*S*-transferases are used for detoxification, it is possible that glutathione depletion might lead to a reduction of the rate of detoxification of mutagenic metabolites formed by CYPs.

Besides methylation, CEs can also be inactivated by glucuronidation and sulfation. If formation of the 4-hydroxyl metabolites is excessive and/or production of the methyl, glucuronide, or sulfate conjugates is insufficient, as the cells are exposed to CEs toxicity, a competitive catalytic oxidation to semiquinones (CE-SQ) and CE-Q can occur. In a second time, CE-SQ and CE-Q may react with glutathione (GSH), catalyzed by ***S***-transferases or with DNA to form stable and depurinating adducts (66, 67).

## Conclusions and perspectives

Because of their beneficial roles in the human gastrointestinal tract, LAB are referred to as ‘probiotics’ and efforts are underway to employ them in modern nutrition habits with so-called functional foods.

Members of *Lactobacillus* and *Bifidobacterium* genera are normal residents of the microbiota in the human gastrointestinal tract, where they developed soon after birth. But, whether such probiotic strains derived from the human gut should be commercially employed in the so-called functional foods is a matter of debate between scientists and the industrial world.

Within a few hours from birth the newborn develops its normal bacterial flora. Indeed human milk frequently contains low amounts of non-pathogenic bacteria like *Streptococcus, Micrococcus, Lactobacillus, Staphylococcus, Corynebacterium* and *Bifidobacterium*.

In general, bacteria start to appear in feces within a few hours of birth. Colonization by *Bifidobacterium* occurs generally within 4 days of life and claims have been made for positive effects of *Bifidobacterium* on infant growth and health.

The aim of the probiotic approach is to repair the deficiencies in the gut flora and restore the protective effect. However, the possible ways in which the gut microbiota is being influenced by probiotics is yet unknown.

To conclude, it is obvious that more research must be done in the near future to clarify the multifactorial association of the CYP (P450) cytochromes’ role in the different diseases’ states, environmental toxic effects or chemical exposures, and nutritional status.
